# Randomized Controlled Trial on the Effects of Home-Based Breathing Exercises on Respiratory Function and Fatigue in COVID-19-Cured Young Patients

**DOI:** 10.3390/healthcare12151488

**Published:** 2024-07-26

**Authors:** Cheol-Hyeon Jeong, Min-Woo Nam, Dong-Yeop Lee, Ji-Heon Hong, Jae-Ho Yu, Jin-Seop Kim, Seong-Gil Kim, Yeon-Gyo Nam

**Affiliations:** 1 Department of Physical Therapy, Sun Moon University, Asan 31460, Republic of Korea; dms6974@naver.com (C.-H.J.); nmw28400@naver.com (M.-W.N.); kan717@hanmail.net (D.-Y.L.); naresa@sunmoon.ac.kr (J.-H.Y.); skylove3373@hanmail.net (J.-S.K.); sgkim4129@sunmoon.ac.kr (S.-G.K.); 2 Digital Healthcare Institute, College of Health Sciences, Sun Moon University, Asan 31460, Republic of Korea; hgh1020@hanmail.net

**Keywords:** COVID-19, pulmonary function, breathing exercise, respiratory muscle, stretching, fatigue

## Abstract

This study investigates the effects of home-based Kakao Healthcare breathing exercises and stretching on respiratory function and fatigue in COVID-19-cured patients. A total of 35 participants performed four movements of home-based breathing exercises and five respiratory muscle stretching exercises four times a week for four weeks. Respiratory function was measured using forced vital capacity(FVC), forced expiratory volume in one second(FEV1), FEV1/FVC ratio, and peak expiratory flow(PEF). Fatigue was assessed using the Fatigue Severity Scale (FSS). Data analysis was performed using independent-sample and paired-sample *t*-tests in SPSS 24, with the significance level set at *p* < 0.05. After four weeks of home-based Kakao Healthcare breathing exercises, there were significant increases in respiratory FVC, FEV1, FEV1/FVC, and PEF function values observed in the training group (T.G.) after the exercise intervention (*p* < 0.05). Such an increase was observed when comparing these values with their corresponding pre-exercise measurements. In contrast, there were no statistically significant differences in respiratory function outcomes before and after exercise in the control group (C.G.) (*p* > 0.05). The FSS scores were statistically significant within the training group (T.G.) (*p* > 0.05). The 4-week Kakao Healthcare breathing exercise scheme was found to be capable of improving some respiratory functions in COVID-19-recovered patients, but it showed no significant improvement in fatigue levels.

## 1. Introduction

The novel coronavirus (COVID-19) emerged in 2019 and primarily spreads through respiratory droplets and close contact. It causes various symptoms including fever, cough, and fatigue, and can lead to severe respiratory issues, particularly in older adults and those with chronic conditions. A significant number of COVID-19 survivors experience long-term symptoms, known as “long COVID”, including reduced lung function and persistent fatigue [1,2]. The virus primarily spreads through respiratory means (especially coughing, sneezing, and close conversation), contaminated surfaces, and biological materials [3]. Clinical symptoms of acute COVID-19 infection include fever, sore throat, cough, fatigue, and gastrointestinal symptoms. Respiratory failure, cardiac damage, and kidney damage can occur in more severe cases. This is particularly common among older individuals and those with underlying chronic conditions. According to one report, around 60 days after the onset of the initial symptoms of COVID-19, only 13% of patients hospitalized for COVID-19 showed no COVID-19-related symptoms, while 32% experienced one or more symptoms. Among them, 55% had three or more symptoms [4,5].

The persistence of symptoms with COVID-19 for more than three months after the first onset of symptoms is called “long COVID” [6]. Recent studies have revealed that more than 60% of COVID-19 survivors experience symptoms not described by an alternative diagnosis [7]. This was found by reduced FVC, FEC1, and FEV1/FEV values compared to the average population. In a meta-analysis combining data from 18 studies, a 1-year follow-up evaluation was conducted on 8591 survivors of COVID-19. The eight most commonly reported symptoms were fatigue/weakness (28%), shortness of breath (18%), joint/muscle pain (26%), depression (23%), anxiety (22%), memory loss (19%), difficulty concentrating (18%), and insomnia (12%). Additionally, there were very few reported cases during the early stages of the COVID-19 pandemic. Among those hospitalized, “severe” symptoms threatening their health were observed. Apart from patients admitted with “severe” COVID-19, it is highly likely that millions of people were infected with SARS-CoV-2 without undergoing official COVID-19 testing and receiving hospital treatment [1,8]. These patients are classified as having “mild” COVID-19, as they only require home care and are expected to recover from the infection [9]. Patients with “mild” COVID-19 may still complain of persistent post-COVID-19 symptoms for weeks after the initial onset [10]. In particular, fatigue (94.9%) and dyspnea (89.5%) were the most common post-COVID-19 symptoms among “mild” patients. This indicates the presence of multiple symptoms and, in turn, unmet healthcare needs among this sample of “mild” and “severe” patients who have recovered from COVID-19 about three months after their initial infection. The phenomenon of symptoms persisting for months after the infection suggests the presence of a “post-COVID-19 syndrome” [11]. In addition, patients with post-COVID-19 symptoms exhibit FVC, FEC1, and FEV1/FEV values that indicate reduced lung function compared to standard groups [12]. 

Cardiopulmonary physical therapy focuses on treating and rehabilitating patients with acute and chronic respiratory diseases according to the guidelines released for physical healers on treating patients with COVID-19 [13]. However, given the highly contagious nature of SARS-CoV-2, a robust respiratory rehabilitation plan must be in place to make optimal use of a limited rehabilitation workforce and reduce risk to health professionals [14]. The effects of respiratory muscle training include increased HPQoL, enhanced exercise resistance, increased strength and endurance of the respiratory muscles, improved lung function, and reduced fatigue and dyspnea [13,15]. According to a recent study, respiratory rehabilitation for elderly patients with COVID-19 can improve their respiratory function and quality of life and reduce anxiety compared to groups without any intervention [16]. Supporting this connection, HBMT (at-home breathing muscle training) for patients with long-term fatigue and dyspnea after COVID-19 led to improved quality of life (QOL) and respiratory function among these patients compared to fake-trained groups [17]. 

Until recently, the apps developed to manage COVID-19 mainly focused on contact tracking, symptom monitoring, and information apps [18]. Nevertheless, recently, remedial education and video guides on the principles of lung rehabilitation and easy exercises to perform at home have been attracting attention and can be used for follow-up monitoring after discharge and for providing guidance to families after discharge [19]. In this regard, remote rehabilitation is being recommended, and recently, a home exercise guidance platform accessed through a remote application was developed in Spain [20]. Completing the recommended exercise process and adhering to these exercises brings about long-term benefits, including reduced pain, improved physical function, and the achievement of agreed goals [21]. Long-term adherence can also significantly improve a patient’s quality of life. It can positively benefit the economy as it can decrease the burden on healthcare systems, as patients can self-manage more effectively [22]. However, a well-established problem when implementing rehabilitation interventions is the adherence rate to home exercise programs [23]. Kakao is a fast-growing software company focused on providing innovative smartphone applications. This company is rooted in the Korean mobile communication market, where customer demands for high-speed connectivity have grown sharply. That is why Kakao programs are so accessible to Koreans [24]. Recently, Kakao created a breathing exercise app to improve the aftereffects of COIVD-19 symptoms. However, until now, no studies have analyzed the effect of this breathing exercise app. This study will examine the relationship between respiratory function and fatigue in COVID-19-cured patients through the “Kakao Healthcare breathing exercises (KBEs)”.

## 2. Materials and Methods

### 2.1. Study Design

**Figure 1:**
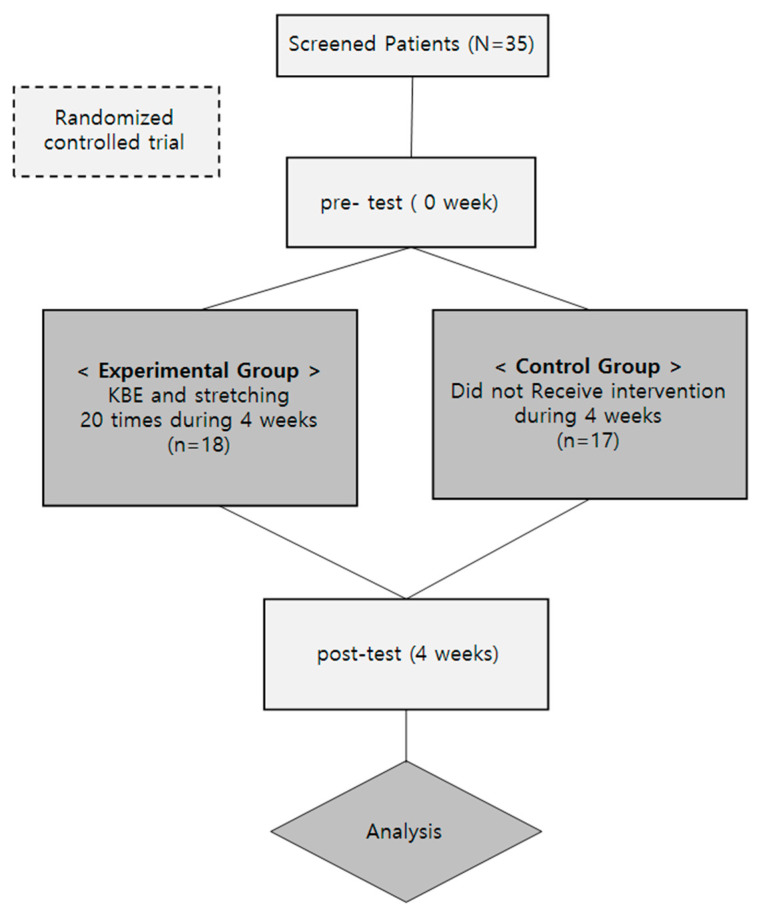
Research procedure.

This study involved an open, randomized controlled trial. A total of 35 participants performed four movements of home-based breathing exercises and five respiratory muscle stretching exercises four times a week for four weeks. The sample size was determined using G*Power software (version 3.1.9.6) under the following configurations: *t*-tests, an effect size corresponding to Cohen’s d for a large effect (0.80), a significance level (α) of 0.05, and a desired statistical power of 0.70. To allow for a possible 10% dropout rate, 17 to 18 participants (out of 35 total participants) were assigned per group. Participants (n = 35) were randomly assigned to either a training group (T.G.) or a control group (C.G.) in a 1:1 ratio. Randomization was stratified by gender and performed using a block-randomization method, i.e., participants were randomly assigned to experimental and control groups using block randomization. Randomization was performed via an Interactive Web Randomization System with Research Randomizer (Research Randomizer Version 4.0; www.randomizer.org accessed on 22 January 2024) and maintained by a third party. The research protocol was approved by the Institutional Review Board (IRB) of Sun Moon University (approval ID: SM-202309-025-3), and this study has been registered at the Clinical Research Information Service (CRIS, KCT0009139; date of registration: 26 January 2024) [Figure 1].

### 2.2. Participants

This study was conducted on 35 COVID-19-recovered young patients, all college students at Sun Moon University in Asan city. All patients gave prior consent before participating in the study. The inclusion criteria were as follows: (1) people aged 19 to 30 years old; (2) people who had been diagnosed with COVID-19, released from isolation, and were experiencing long COVID symptoms; (3) people for whom ≥ six months had passed since the onset of any other acute diseases. The exclusion criteria were as follows: (1) people with moderate or severe heart disease; (2) people with severe ischemic or hemorrhagic stroke or a neurodegenerative disease; (3) people with visual, auditory, or vestibular problems; (4) people who had other respiratory conditions.

### 2.3. Measurement Equipment

#### 2.3.1. Measuring Lung Capacity (Pulmonary Function)

We used a desktop spirometer (Model et al., COSMED, Rome) for assessing respiratory function. The following parameters related to respiratory function were measured: (1) Forced Expiratory Volume in 1 s (FEV1); (2) Forced Vital Capacity (FVC); (3) Forced Expiratory Volume in 1 s/Forced Vital Capacity (FEV1/FVC); (4) Peak Expiratory Flow (PEF) (Figure S1).

#### 2.3.2. Measuring Fatigue (Fatigue Severity Scale)

The post-COVID-19 patients’ fatigue was evaluated through an evaluation tool that quantified their perceived exertion. FFS is a 9-item scale that measures the severity of fatigue and its effect on a person’s activities and lifestyle in patients with various disorders. It was initially devised for people with multiple sclerosis (MS) or systemic lupus erythematosus. It includes nine items, each consisting of a statement for which respondents are asked to indicate their level of agreement from 1 (strongly disagree) to 7 (strongly agree). An assessment of the two groups before and after the intervention was conducted using the FSS [25,26].

### 2.4. Experimental Procedures 

The participants (T.G.) were trained in breathing exercises to carry out at home. This consisted of 2 sections: (1) stretching exercises and (2) Kakao Healthcare breathing exercises (Jeju-si, Kakao, Republic of Korea Kakao). They performed the breathing exercises at home for four weeks (10 min/day for four days/week). They were asked to practice these exercises separately at home during the experiment. To ensure that the subjects could follow the program correctly, an experimenter taught them the program until they could exercise on their own. They received a video clip of the exercises that could be opened and followed via a smartphone. Also, through a Kakao chat or phone call, they were reminded to follow the exercise routine four times a week. The experimental group recorded their progress after completing the exercise, and the experimenters checked the progress once a week. Among the participants, individuals who did not achieve at least 80% compliance with the exercise scheme were excluded from the experiment. Measurements were conducted a total of two times: once before the start of the experiment and again after the 4-week intervention. Pre-tests were conducted within 24 h before the start of the experiment, and post-tests were conducted within 24 h after the last investigation (Figure S2).

#### 2.4.1. Application of Breathing Exercises

The “KBE” scheme was developed to improve symptoms such as coughing, phlegm, and chronic fatigue, known as aftereffects, with a series of mild breathing exercises falling under the slogan “COVID-19, let us rest satisfactorily and rest well”. This consists of four exercises: exercises to relieve coughing and phlegm and exercises to recover from fatigue. The KBEs refer to the guidelines on the aftereffects of COVID-19 presented by the PASC Dashboard of the American Society of Rehabilitation Medicine. In addition, the KBE app shows exercise guidance videos to make it easier to follow the exercises. It guides you through exhalation, inspiration, and expansion time, and you can enjoy the process by listening to the sounds of your breathing and the app together.

The “balloon breathing exercise” is similar to “pursed-lips breathing”. Regular breathing is performed once at the beginning and end of this exercise. While following the breathing exercise video, you take a 3 s inhalation and a 1 s expansion, puffing out the cheeks as you exhale. This process is repeated four times, and the total duration is 1 min and 28 s. Pursed-lips breathing involves inhaling through the nose and exhaling through pursed lips, which helps to reduce breathlessness, decrease the respiratory rate, and minimize dynamic hyperinflation during exercise training with the aim of overall endurance improvement. Additionally, supplemental oxygen at 20% has been successfully used during exercise training to alleviate the respiratory muscle workload [27,28]. 

The “hahaha breathing exercise” technique helps with mucus clearance and is similar to the “huff cough” method. Normal breathing is performed once at the beginning and end of this exercise. While following the breathing exercise video, you inhale once and then exhale with a “ha~” sound three times. This process is repeated four times, and the total duration is 1 min and 28 s. Using forced expiratory maneuvers like the huff cough can help propel secretions. Huff coughing is performed as an open-glottis maneuver that utilizes forced expiration, enabling the movement of mucus by dynamically compressing the same pressure points in the airway, thus increasing the linear velocity of the expiratory flow. When initiating forced expiration at lower lung volumes, the isobaric points move toward the periphery and smaller airways. Forced expiration at higher lung volumes, on the other hand, shifts the isobaric points centrally towards the larger central airways [29]. 

The “heub heub heub breathing exercise” helps to increase lung capacity. Regular breathing is performed once at the beginning and end of this exercise. While following the breathing exercise video, you inhale three times to make the “heub~” sound and then exhale. This process is repeated four times, and the total duration is 1 min and 32 s.

The “heub ha heub ha breathing exercise” helps with physical activation. Regular breathing is performed once at the beginning and end of this exercise. While following the breathing exercise video, you inhale rapidly to make the “heub” sound and then exhale rapidly to make the “ha” sound, following the speed demonstrated in the instructional video. This process is repeated six times, and the total duration is 1 min and 12 s (Figure S3).

#### 2.4.2. Stretching Exercise

Respiratory muscle stretching was carried out under the guidance of a rehabilitation therapist. RMSG consists of five sections [30]:

Pattern 1—“Elevating and pulling back the shoulders”: As you inhale deeply through your nose, slowly raise and lower both shoulders. After taking a deep breath, exhale slowly through your mouth, relax, and lower your shoulders.

Pattern 2—“Stretching the upper chest”: Place both hands on your upper chest. Pull back your elbows and pull down your chest while lifting your chin and inhaling deeply through your nose. Exhale slowly through your mouth and relax.

Pattern 3—“Stretching the back muscle”: Place your hands in front of your chest. Inhale slowly through your nose, gently stretching your back and moving your hands back and forth smoothly. After taking a deep breath, exhale slowly and return to your original position.

Pattern 4—“Stretching the lower chest”: Hold the edges of a face towel with both hands extended to shoulder height. After taking a deep inspiration, slowly exhale and move your arms upward. After taking a deep breath, lower your hands and breathe normally.

Pattern 5—“Elevating the elbow”: Grip one hand behind your head. Take a deep breath through your nose. While gradually exhaling through your mouth, stretch your trunk by raising your elbow as tall as is easily possible. Return to the original position while breathing normally (Figure S4).

### 2.5. Data Analysis

Statistical data analysis was performed with SPSS version 24.0. In this study, normality was verified through Shapiro–Wilk verification. The Chi-square test was used to compare the study groups for the categorical variables. The intra-group data were compared with a paired *t*-test. The inter-group comparison of the improvement (differences in pre–post values) between groups was evaluated with an independent-sample *t*-test. The statistical significance level was set to the 95% confidence level.

## 3. Results

A total of 35 patients were assessed for eligibility. There were no statistically significant differences between the two groups of patients [Table 1]. 

Inter-group analysis showed that the training group (T.G.) had significantly higher FEV1 (3.88 ± 1.13 vs. 4.37 ± 1.00, *p* = 0.002), FEV1/FVC% (75.16 ± 10.71 vs. 84.46 ± 6.45, *p* = 0.005), and PEF (7.74 ± 2.79 vs. 8.92 ± 2.50, *p* = 0.003) values compared to the control group (C.G.). There was no significant difference in FVC (5.08 ± 1.15 vs. 5.16 ± 1.10, *p* = 0.181). The lung function parameters FVC (4.99 ± 0.95 vs. 4.93 ± 0.99, *p* = 0.456), FEV1 (3.94 ± 0.88 vs. 3.95 ± 0.92, *p* = 0.268), FEV1/FVC% (78.17 ± 9.28 vs. 78.64 ± 9.34, *p* = 0.418), and PEF (7.12 ± 2.78 vs. 7.64 ± 2.90, *p* = 0.067) in the C.G. did not show any significant differences. The T.G. and the C.G. were compared after four weeks of respiratory rehabilitation in terms of lung function parameters. There was a statistically significant difference found between the two groups in FEV1 (0.491 ± 0.568 vs. 0.124 ± 0.451, *p* = 0.010) and FEV1/FVC% (9.30 ± 12.189 vs. 0.471 ± 9.227, *p* = 0.022). However, there was no statistically significant difference between FVC (0.083 ± 0.227 vs. −0.055 ± 0.364, *p* = 0.181) and PEF (1.176 ± 1.462 vs. 0.520 ± 1.359, *p* = 0.179) [Table 2].

Regarding the fatigue scores, there was no statistically significant difference found between the two groups (*p* = 0.125). However, inter-group analysis revealed that there was a statistically significant difference within the T.G. participants (*p* = 0.032), and the C.G. showed no significant differences (*p* = 0.755) [Table 3].

## 4. Discussion

This study focuses on the medical challenges faced by COVID-19-recovered patients with mild to moderate symptoms who were isolated and did not require hospitalization. Our findings suggest that home-based breathing exercises combined with stretching can significantly improve respiratory function, particularly FEV1 and FEV1/FVC%, but have limited effects on fatigue. These results are consistent with previous studies showing the benefits of respiratory rehabilitation in improving lung function [15,16,17].

Previous studies reported that lung function parameters, including FVC, FEV1, and FEV1/FVC(%), improved over time when comparing between experimental and control groups. However, no significant differences were found between the adjusted groups. In this study, similar improvements in lung function parameters, excluding FVC within the groups, were observed over time. However, significant differences were found between the groups regarding FEV1 and FEV1/FVC (%). A possible explanation for this could be related to the trend of shorter tracking periods in the clinical trials that have reported the most significant benefits of digital interventions, potentially resulting in decreased revenue as the usage period lengthens. There are various reasons for a decline in exercise adherence over time, but it is primarily confirmed that exercise motivation decreases as time passes [31]. Furthermore, unlike the exercise regimen in the aforementioned previous paper that included only bracing exercises, another recent study incorporated exercises at a higher level of intensity, including lower-limb muscle strength training and aerobic exercises. However, the post-COVID-19 rehabilitation program recommends conducting moderate-intensity exercises, prioritizing safety, instead of low-intensity exercises. Finally, it emphasizes that pulmonary rehabilitation programs can vary in effectiveness based on individual needs and functional impairments, which is relevant in this context [32,33]. 

According to a recent meta-analysis, it has been found that 32% (n = 25,268) of post-COVID-19 individuals experience fatigue for more than 12 weeks after recovery [34]. Unlike other persistent symptoms such as dehydration, cough, and weakness, fatigue persists and worsens over time. The etiology of such fatigue remains unclear, but it is known to be complex and multifactorial in its mechanistic aspects. Clear immunological findings related to fatigue have not been observed in COVID-19 patients. Therefore, non-pharmacological interventions are recommended for treating fatigue in these patients [35]. This study’s Fatigue Severity Scale scores significantly decreased only in the experimental group (*p* = 0.032). The between-group effect size showed a marginal reduction in fatigue severity after the intervention (*p* = 0.125). A recent study reported a significant reduction in fatigue severity when breathing exercises accompanied by chest expansion and arm movements were conducted for six weeks: despite the non-significant difference between the baseline and the 2-week mark, there was a significant difference between the baseline and 6-week measurements [36]. Through this study, it was observed that four weeks of breathing exercises also had an effect on reducing fatigue severity. 

The KBE scheme in this study incorporates various breathing techniques, including respiratory control, pursed-lips breathing, and diaphragmatic breathing. These deep breathing techniques can benefit anyone, but they play a particularly crucial role in the recovery process of individuals with COVID-19. These exercises can be performed at home during self-isolation and easily incorporated into daily life. Deep-breathing exercises have shown the potential to reduce anxiety and stress, which are commonly experienced by individuals who have had severe symptoms or have been hospitalized. These breathing exercises can help to recover diaphragmatic function and enhance lung capacity. Our primary goal is to enhance people’s ability to practice deep breathing during resting and all activities [37]. Furthermore, pursed-lips breathing involves inhaling through the nose and exhaling through pursed lips. This technique aims to reduce airway collapse, decrease the respiratory rate, and improve endurance during exercise training [27]. Recent research has demonstrated that oxygen supplementation through pursed-lips breathing can help reduce the workload on respiratory muscles during exercise training [38]. Finally, stretching techniques, which included stretching of the neck, upper chest, pectoral muscles, and lateral chest and mobilization of the posterior joints, were found to increase lung compliance by up to 50 mL [39,40]. Our study showed a significant improvement in the T.G. (treatment group) after four weeks of respiratory rehabilitation training. These results may be attributed to the inclusion of various respiratory muscles, including the diaphragm, intercostal muscles, and abdominal muscles, in our respiratory rehabilitation training plan. These muscles play a crucial role in maintaining respiratory function. Functional deficits in these muscles can lead to breathlessness, paradoxical breathing, increased extension of the chest muscles during respiration, and reduced chest wall movement during respiration. Slower breathing rates lead to reduced energy consumption, enhanced lung ventilation, and improved blood oxygenation levels. From a clinical perspective, although the results of this study may not have shown that home-based bracing exercises are more effective than standard rehabilitation therapy, it does suggest that home-based bracing exercises can be used as an effective intervention for improving respiratory parameters in patients with post-acute COVID-19. Furthermore, it is worth noting that older patients are more likely to choose inpatient rehabilitation. In comparison, younger patients are inclined towards outpatient rehabilitation due to its better compatibility with their home or work commitments. Especially for a significant portion of outpatient rehabilitation patients, it is crucial to establish personalized home-based rehabilitation plans or transition them to long-term rehabilitation phases at outpatient centers. This approach promotes physical activity and helps these individuals to maintain a healthy lifestyle [21].

## 5. Conclusions

The 4-week home-based breathing exercises (KBEs) and stretching program significantly improved pulmonary function (FEV1, FEV1/FVC%, PEF) in COVID-19-recovered patients, particularly in FEV1 and FEV1/FVC%, when compared to the control group. However, no significant improvements in fatigue levels were observed. These findings suggest that home-based breathing exercises can be used as an effective intervention for improving lung function in COVID-19 patients.

## 6. Limitations

This study acknowledges the evidence for the effectiveness of home-based KBEs and stretching programs but recognizes several limitations that should be considered. Firstly, this study did not show significant improvements in pulmonary respiratory function (FEV, PEF, FVC) after the intervention, possibly due to the relatively short duration of the respiratory rehabilitation period. Secondly, this review could not provide adequate evidence for the effects of breathing exercises on patients with severe lung injury (mild to moderate lung injury) and the long-term (>1 year) effects of breathing exercises in the these patients due to the included population. Thirdly, due to data limitations, this review could not evaluate the effects of breathing exercises on comorbidities after COVID-19. For this reason, caution is needed in interpreting and generalizing the results of this review. Sixthly, measurements of respiratory muscle function and chest movements were not conducted, which prevented the assessment of changes in fatigue related to these factors. Fourthly, this study relied on participants’ self-discipline for exercise progress. Therefore, consideration should be given to whether the exercise intervention was effectively implemented. Fifth, as far as we know, there is no breathing exercise program like the KBS program. Therefore, there may be a need for more evidence on the KBS scheme. Finally, the wide variation in COVID-19 confirmation dates makes distinguishing between the acute and chronic phases challenging. Despite these limitations, this study has several notable strengths. Firstly, it contributes to supporting the use of home-based exercise programs, which are especially valuable during times when access to healthcare facilities is limited, such as due to the ongoing pandemic and its effects. Secondly, this study provides valuable insights into the feasibility and potential benefits of KBEs and stretching programs for pulmonary rehabilitation, which can inform future research and clinical practice.

## Figures and Tables

**Table 1:** Baseline characteristics for COVID-19 patients: intervention group versus control group.

Characteristic	T.G. (n = 18)	C.G. (n = 17)	*p*-Value
Male, n (%)	13 (72.2)	12 (66.7)	0.91
Age, years (M ± SD)	22.2 (1.67)	21.4 (1.3)	0.12
Height, cm (M ± SD)	170.8 (9.11)	171.3 (8.3)	0.87
Weight, kg (M ± SD)	65.7 (12.56)	72.5 (16.0)	0.16
Smoking habit, n (%)	1 (5.6)	3 (16.7)	0.27
Time from COVID-19 diagnosis, months (M ± SD)	12.3 (6.09)	13.2 (7.92)	0.68

**Table 2:** Comparison of lung function between the two groups before and after the intervention.

Parameter	T.G. (n = 18)			C.G. (n = 17)			Between-Group (Over 4 Weeks) (*p*)
	Pre (Mean ± SD)	Post (Mean ± SD)	Intra-Group (*p*)	Pre (Mean ± SD)	Post (Mean ± SD)	Intra-Group (*p*)	
FVC (L)	5.08 ± 1.15	5.16 ± 1.10	0.138	4.99 ± 0.95	4.93 ± 0.99	0.456	0.181
FEV1 (L)	3.88 ± 1.13	4.37 ± 1.00	0.002	3.94 ± 0.88	3.95 ± 0.92	0.268	0.010
FEV1/FVC%	75.16 ± 10.71	84.46 ± 6.45	0.005	78.17 ± 9.28	78.64 ± 9.34	0.418	0.022
PEF (L)	7.74 ± 2.79	8.92 ± 2.50	0.003	7.12 ± 2.78	7.64 ± 2.90	0.067	0.179

**Table 3:** Comparison of FSS scores between the two groups before and after the intervention.

Parameter	T.G. (n = 18)			C.G. (n = 17)			Between-Group (Over 4 Weeks) (*p*)
	Pre (Mean ± SD)	Post (Mean ± SD)	Intra-Group (*p*)	Pre (Mean ± SD)	Post (Mean ± SD)	Intra-Group (*p*)	
FSS	4.05 ± 1.33	3.85 ± 1.03	0.032	3.32 ± 1.23	2.95 ± 1.59	0.755	0.125
Item 1	5.56 ± 1.54	3.78 ± 2.10	0.003	4.41 ± 1.41	4.06 ± 1.43	0.422	0.088
Item 2	4.28 ± 2.94	3.28 ± 1.77	0.029	3.94 ± 1.63	3.65 ± 1.61	0.492	0.538
Item 3	4.33 ± 1.64	2.94 ± 1.62	0.004	3.53 ± 1.94	3.24 ± 1.78	0.584	0.243
Item 4	4.50 ± 1.65	3.17 ± 1.58	0.009	3.59 ± 1.93	3.12 ± 1.83	0.280	0.300
Item 5	3.94 ± 1.58	2.83 ± 1.61	0.022	2.76 ± 1.71	2.88 ± 1.65	0.805	0.182
Item 6	3.24 ± 1.60	2.65 ± 1.22	0.086	2.47 ± 1.46	2.82 ± 2.12	0.502	0.329
Item 7	3.44 ± 1.65	2.83 ± 1.42	0.238	2.47 ± 1.50	2.53 ± 1.97	0.899	0.649
Item 8	3.89 ± 1.84	3.11 ± 1.60	0.012	3.88 ± 1.69	3.82 ± 1.87	0.914	0.488
Item 9	3.50 ± 1.85	2.33 ± 1.13	0.006	2.65 ± 1..80	2.64 ± 1.80	1.00	0.206

## Data Availability

The data used to support the findings of this study are available from the corresponding author upon reasonable request.

## Supplementary Materials

The following supporting information can be downloaded at https://www.mdpi.com/article/10.3390/healthcare12151488/s1: Figure S1: Spirometer measurement; Figure S2: Pulmonary functional test; Figure S3: Kakao Healthcare breathing exercises; Figure S4: Stretching exercises.
