# Supplementary material for: Randomized Controlled Trial on the Effects of Home-Based Breathing Exercises on Respiratory Function and Fatigue in COVID-19-Cured Young Patients
Source: Healthcare (Basel). 2024 Jul 26;12(15):1488. doi: 10.3390/healthcare12151488 (PMC11311616; doi:10.3390/healthcare12151488)
Supplement: Supplementary file 1 [file healthcare-12-01488-s001.zip › healthcare-3111083-supplementary.pdf]

Figure S1 : Spirometer

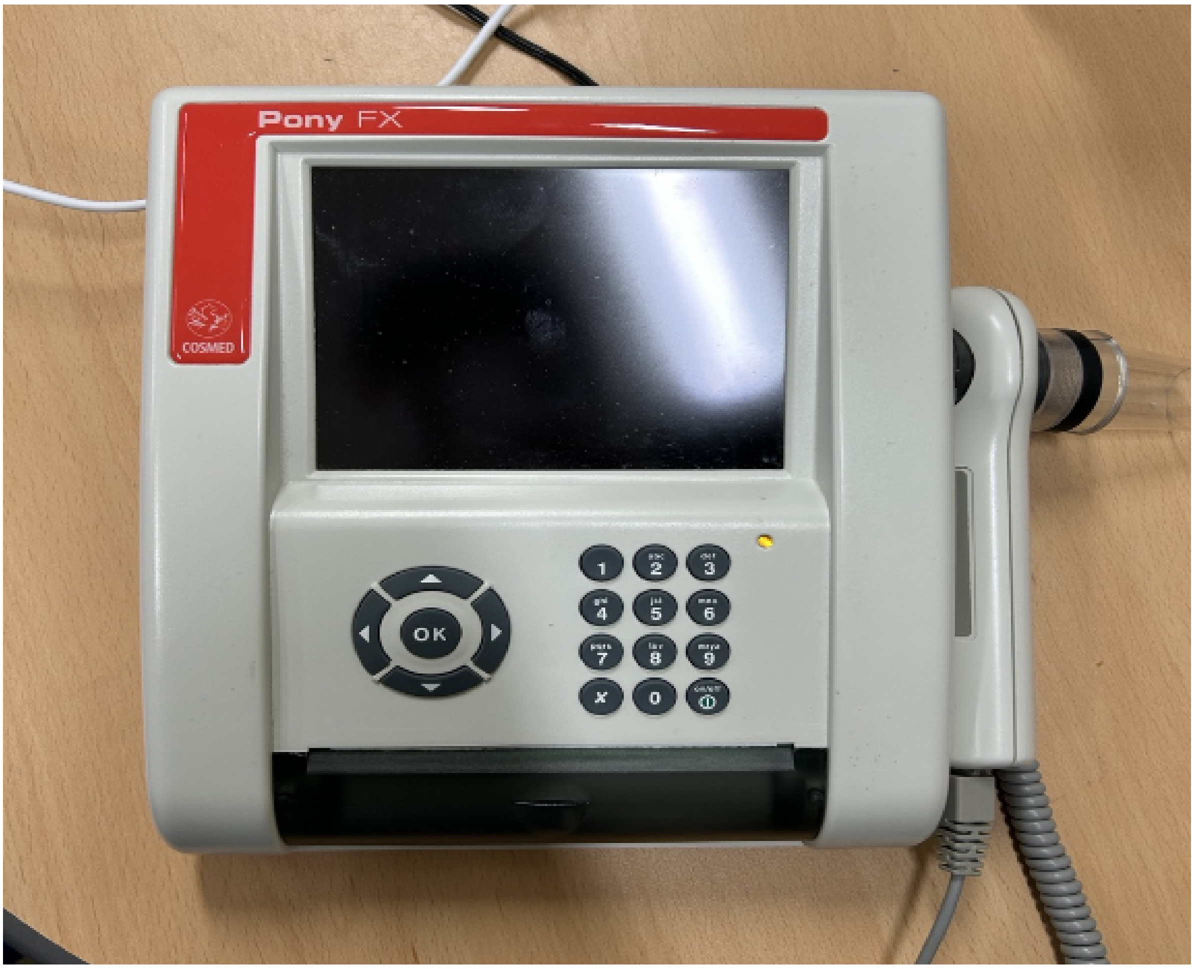

Figure S2 : Pulmonary functional Test

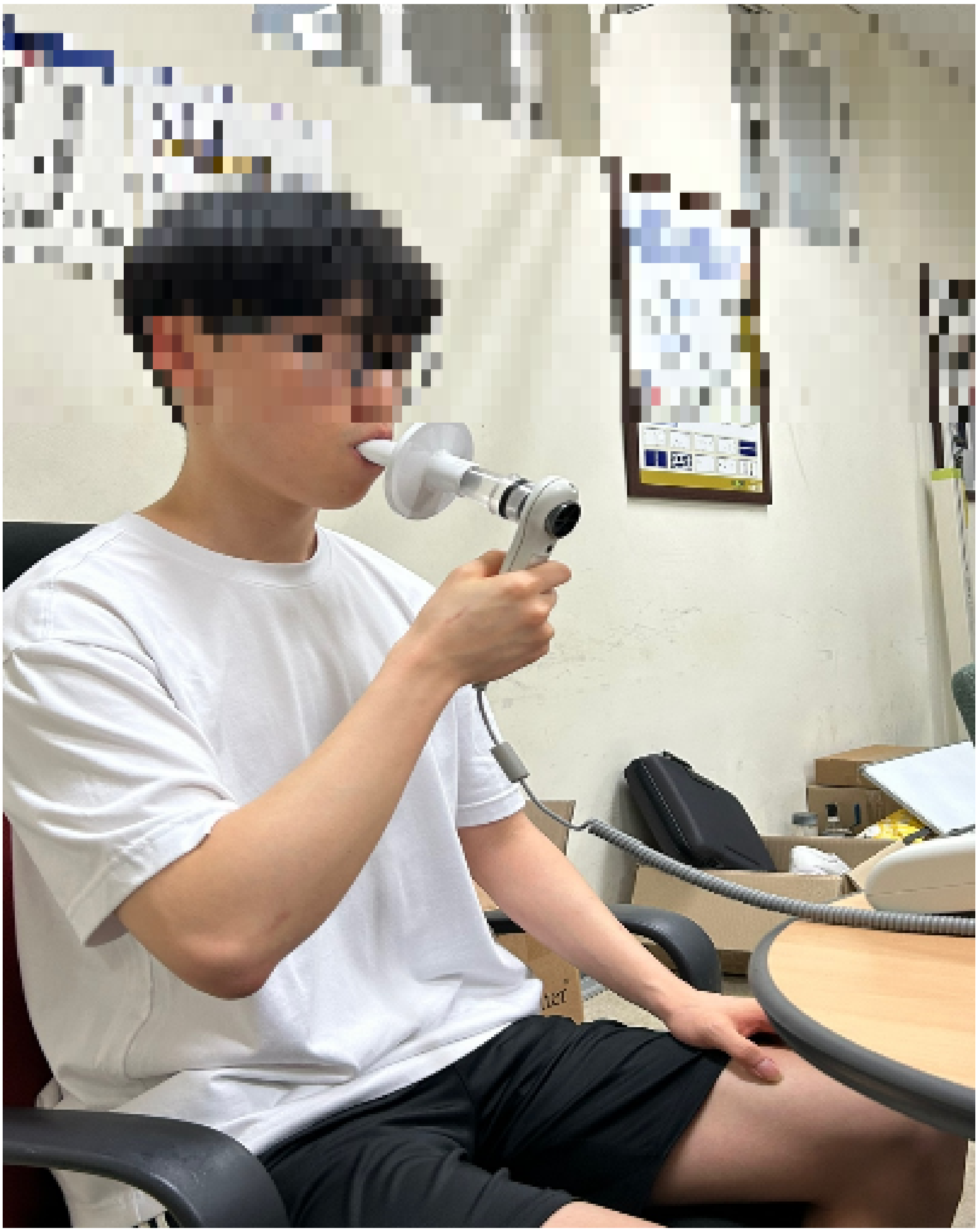

Figure S3 : Kakao Health Care Breathing Exercise

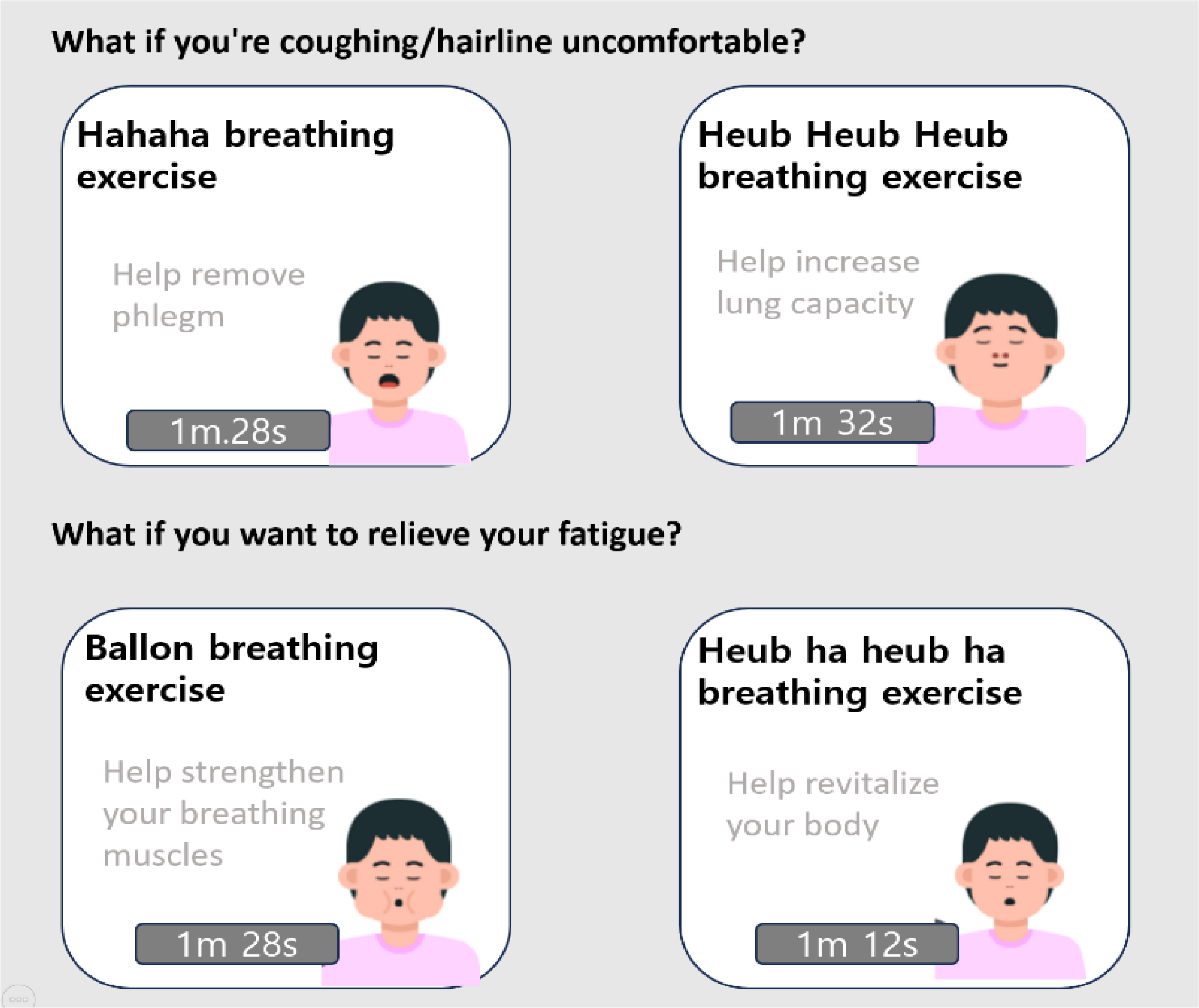

Figure S4 : Stretching exercies

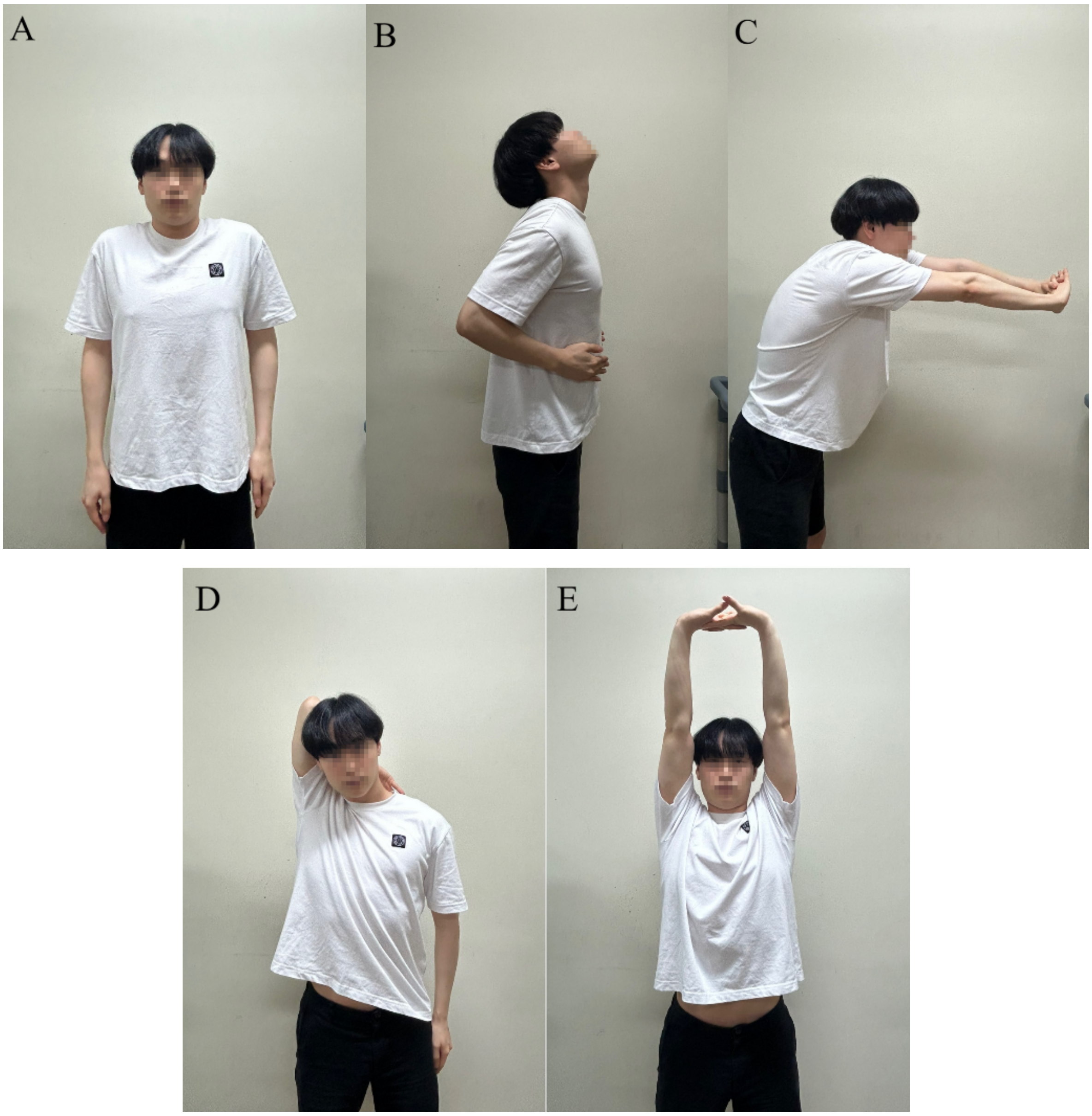

A: Elevating and pulling back theshoulders; B: Stretching the upper chest; C: Stretching the backmuscle; D:Elevating the elbow Stretching; E: Stretching the lower chest
